# Novel Mutations in ACP5 and SAMHD1 in a Patient With Pediatric Systemic Lupus Erythematosus

**DOI:** 10.3389/fped.2022.885006

**Published:** 2022-05-13

**Authors:** Soon-Min Hong, Wei Chen, Jiaqi Feng, Dai Dai, Nan Shen

**Affiliations:** ^1^Shanghai Institute of Rheumatology, School of Medicine, Renji Hospital, Shanghai Jiao Tong University, Shanghai, China; ^2^Department of Rheumatology, School of Medicine, Renji Hospital, Shanghai Jiao Tong University, Shanghai, China; ^3^Department of Pediatrics, School of Medicine, Renji Hospital, Shanghai Jiao Tong University, Shanghai, China; ^4^Shenzhen Futian Hospital for Rheumatic Diseases, Shenzhen, China; ^5^State Key Laboratory of Oncogenes and Related Genes, Shanghai Cancer Institute, School of Medicine, Renji Hospital, Shanghai Jiao Tong University, Shanghai, China; ^6^Center for Autoimmune Genomics and Etiology (CAGE) and Divisions of Rheumatology, Cincinnati Children's Hospital Medical Center, Cincinnati, OH, United States; ^7^Joint Research Laboratory for Rheumatology of Shenzhen University Health Science Center and Shenzhen Futian Hospital for Rheumatic Diseases, Shenzhen, China

**Keywords:** novel mutation, ACP5/TRAP, SAMHD1, systemic lupus erythematosus (SLE), whole-exome sequencing (WES), bioinformatics

## Abstract

**Background:**

The study of genetic predisposition to pediatric systemic lupus erythematosus (pSLE) has brought new insights into the pathophysiology of SLE, as it is hypothesized that genetic predisposition is greater in children. Furthermore, identifying genetic variants and linking disrupted genes to abnormal immune pathways and clinical manifestations can be beneficial for both diagnosis and treatment. Here, we identified genetic alterations in a patient with childhood-onset SLE and analyzed the immunological mechanisms behind them to support future diagnosis, prognosis, and treatment.

**Methods:**

Whole exome sequencing (WES) was adopted for genetic analysis of a patient with childhood-onset SLE. Gene mutations were confirmed by Sanger sequencing. Clinical data of this patient were collected and summarized. Ingenuity Pathway Analysis was used to provide interacting genes of the perturbed genes. Online Enrichr tool and Cytoscape software were used to analysis the related pathways of these genes.

**Results:**

We present a case of a 2-year-old girl who was diagnosed with idiopathic thrombocytopenic purpura (ITP) and SLE. The patient was characterized by cutaneous bleeding spots on both lower extremities, thrombocytopenia, decreased serum complements levels, increased urinary red blood cells, and positive ANA and dsDNA. The patient was treated with methylprednisolone and mycophenolate, but clinical remission could not be achieved. The genomic analysis identified three novel mutations in this pSLE patient, a double-stranded missense mutation in ACP5 (c.1152G>T and c.420G>A) and a single-stranded mutation in SAMHD1 (c.1423G>A). Bioinformatic analysis showed that these two genes and their interacting genes are enriched in the regulation of multiple immune pathways associated with SLE, including cytokine signaling and immune cell activation or function. Analysis of the synergistic regulation of these two genes suggests that abnormalities in the type I interferon pathway caused by genetic variants may contribute to the pathogenesis of SLE.

**Conclusion:**

The combined complexity of polymorphisms in the coding regions of ACP5 and SAMHD1 influences the susceptibility to SLE. Alterations in these genes may lead to abnormalities in the type I interferon pathway. Our study extends the spectrum of mutations in the ACP5 and SAMHD1 genes. The identification of these mutations could aid in the diagnosis of SLE with genetic counseling and suggest potential precise treatments for specific pathways.

## Introduction

Systemic lupus erythematosus (SLE) is a chronic multisystem autoimmune disease characterized by the production of autoantibodies and overactivation of the type I interferon (IFN) pathway, with alternating exacerbations and remissions (1). SLE is a heterogeneous disease and the diagnosis of SLE requires the presence of 4 of the 11 defining criteria. Clinical manifestations of SLE patients include arthritis, rash, serositis, various cytopenias, renal disease, psychiatric, neurological, and other manifestations (2, 3). Despite the wide variability, approximately 50% of SLE patients have persistently elevated blood levels of type I IFN over time. In addition, 60–80% of SLE patients have increased expression of interferon-stimulated genes (ISG) in peripheral blood, which is referred to as an IFN signature (1, 4). As with other heterogeneous diseases, the pathogenesis of SLE remains unclear. However, heritability has long been recognized as an important causative factor, affecting approximately 66% in SLE (5). To date, genome-wide association studies (GWAS) have identified more than 80 loci closely associated with lupus, and single-gene susceptibility to SLE has been suggested to be caused by single-nucleotide mutations in the coding regions of nearly 30 genes (6–8). The current view linking many susceptibility genes to key immune pathways is consistent with previous experimental studies including the role of immune complexes, host immune signaling, and interferon pathways in the pathogenesis of SLE (8, 9). Monogenic type I interferon diseases such as spinal chondrodystrophy (SPENCD) and Aicardi-Goutières syndrome (AGS) clinically overlap with SLE, both of which are associated with spontaneous type I IFN responses with no detectable exogenous viral infection (10). SPENCD is driven by an autosomal recessive mutation in ACP5. AGS can have different patterns of inheritance, mostly caused by single mutations in the ADAR, TREX1, RNASEH2A, RNASEH2B, RNASEH2C or SAMHD1 genes (11). These studies, together with laboratory observations, suggest that IFN plays an important role in the pathogenesis of SLE.

Given the complexity and heterogeneity of SLE, identifying genetic alterations and linking them to underlying immunologic pathogenesis could improve individualized diagnostic and therapeutic approaches to SLE. Here, we identified a pediatric-onset SLE patient with germline mutations in both ACP5 and SAMHD1.This patient carried three novel mutations from both father and mother, resulting in a double missense mutation in ACP5 (c.1152G>T in the mother and c.420G>A in the father) and a single missense mutation in SAMHD1 (c.1423G>A in the mother). We found that these two genes and their regulatory networks were enriched in multiple immune pathways associated with SLE, including cytokine signaling and immune cell activation or function. We also analyzed the synergistic regulation of these two genes and demonstrated their combined contribution to type I IFN, which suggests a pathogenic role of this genetic variant in this pSLE patient.

## Materials and Methods

### Human Patients

We collected and summarized the clinical data of a patient who was diagnosed with lupus and carried specific gene mutations. Laboratory results before treatment were recorded for analysis. Written informed consent to participatein this study was provided by the participants' legal guardian/next of kin. Protocol of this study was approved by the Renji Hospital Biobank is funded by the National Human Genetic Resources Sharing Service Platform (2005DKA21300).

### DNA Sequencing

DNA from probands and their family members were isolated and purified from blood and prepared for whole-exome sequencing (WES). DNA samples were enriched with Human SureSelect XT2 All Exon V4 Kit and sequenced by Illumina HiSeq 2000 (Illumina, Inc.). WES had 21% low or uncovered exon bases. Bioinformatic analysis was performed at JCSMR, ANU. Raw sequence reads were aligned to the reference genome (Hg19) and single-nucleotide variants and small insertions and deletions called using GATK. All SNVs of interest in ACP5 and SAMHD1 were confirmed by Sanger sequencing. Amplifluor to detect ACP5G290V, ACP5R46Q, and SAMHD1R408H in the APOSLE cohort was performed using the CHEMICON Amplifluor SNPs HT Genotyping System Fam-Joe kit S7909 (Merck-Millipore). The 45 and Up (12, 13) and ASPREE (14) datasets were used as reference healthy controls, accessed through the MGRB Collaborative (http://sgc.garvan.org.au/mgrb/initiatives).

### WES Data Processing and Batch Correction

Probes were filtered out if the detection P value was greater than 0.01 for at least 100% of the samples. All data values <10 was set to 10 and then the data were log2 transformed. An additional filter selecting the 75% most variable transcripts was performed, leaving a total 18,004 probes for analysis. Principal variance component analysis (PVCA) was conducted to identify undesirable sources of technical variability within the data and batch correction was applied to correct for this technical variation. Both PVCA and batch correction were conducted using JMP Genomics 7.0 (SAS Institute) analysis software.

### Protein Preparation and Mutations Interactions Analysis

The X-ray resolved crystal structures of wild type ACP5 (PDB: 2BQ8) and SAMHD1 (PDB: 7A5Y) were download from RCSB PDB database, the mutations' structures were generated by pymol2.2. Water molecules, ions, heteroatoms, and all ligands were eliminated. Finally, the protonation states of the wild and mutated structures were then deliberated using a H++ server. Additionally, all missing hydrogen atoms were inserted by pymol2.2 The interactions analysis was used pymol2.2.

### Statistical and Bioinformatics Analysis With IPA, Cytoscape

We used BioProfiler searches the Ingenuity Pathway Analysis (IPA, Qiagen) database to explore associations gene and phenotypes. IPA was used to construct pathways and networks (15, 16), to quickly identify biological relationships, mechanisms, pathways, functions and diseases most relevant to experimental datasets. Then we obtain two comprehensive lists of proteins implicated in either the ACP5 or SAMHD1 by IPA. Detailed information of the expressed proteins and endogenous biochemical compounds that have been associated with genes by using the “Annotation” module in IPA. The protein-protein interaction (PPI) network based on ACP5 and SAMHD1 was obtained via String (V.11.5) to predicting the protein interactions and obtaining protein connection scores, and the interaction scores beyond 0.400 were uploaded to Cytoscape for visualization and analysis of biological networks (17, 18). Protein overlapping network identification by using CentiScaPe to investigate node centrality in both networks, and important module in the protein network related to the hub genes were abstracted by using Molecular Complex Detection (MCODE) (19). The Kyoto Encyclopedia of Genes and Genomes (KEGG) pathway analysis from ClueGO were used to perform biological pathway enrichment analysis for the core cluster identified by MCODE (20).

## Results

### Case Presentation

**Figure 1:**
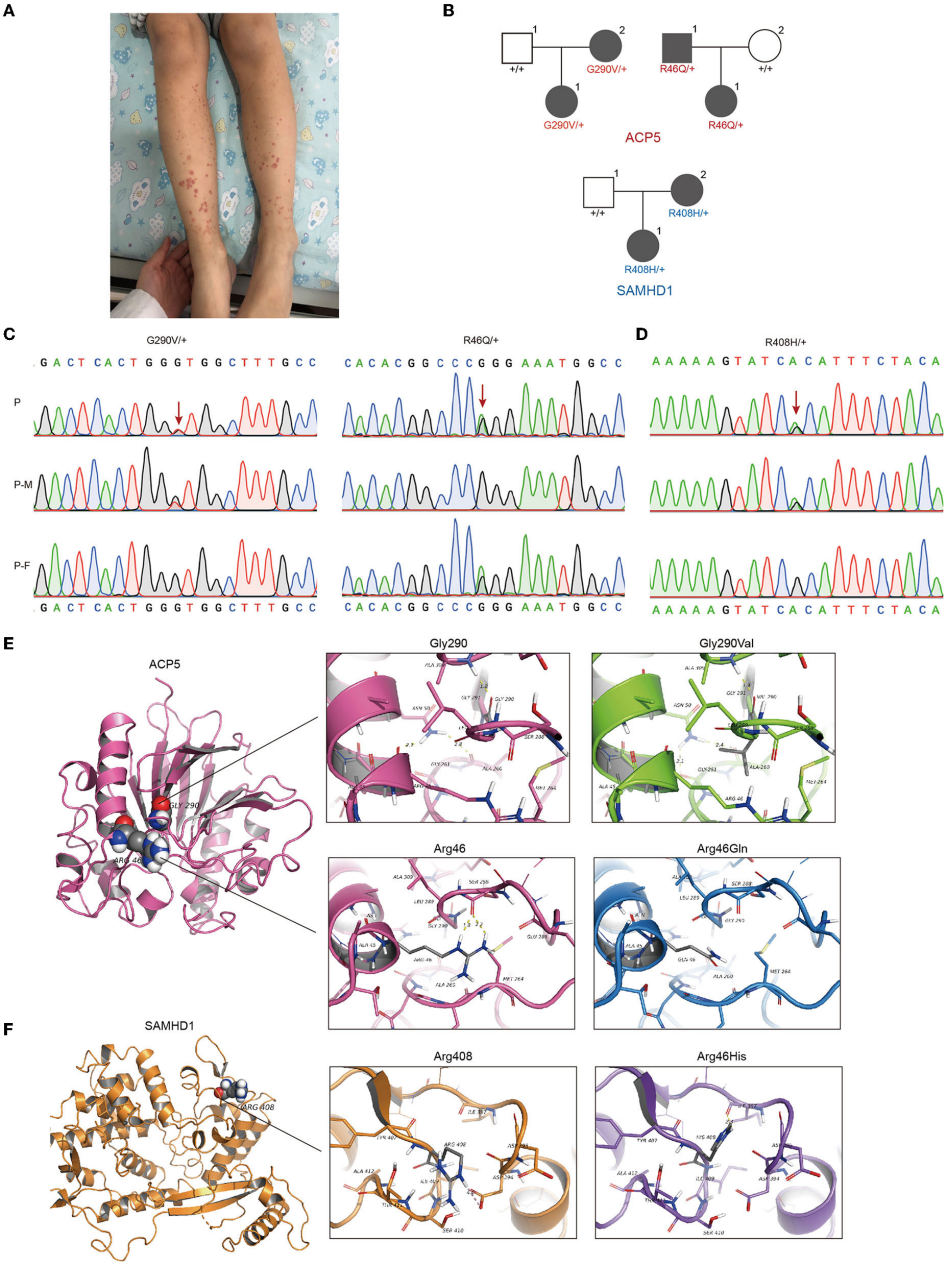
Identification of gene mutations in patient with pSLE. **(A)**The patient initially presented skin bleeding spots in both lower limbs. **(B)** Pedigrees showing affected probands and parents. Sanger sequencing confirmation of the c.1152G>T (p.Gly290Val), c.420G>A (p. Arg46Gln) at ACP5 **(C)** and c.1423G>A (p.Arg408His) at SAMHD1 **(D)** variant identified in our patient using exome sequencing. Chromatograms represent the reference (top) and the mutant sequence. The variant is indicated by the red arrow. **(E)** Top: Structure modeling of wild type and p.Gly290Val mutation of ACP5. Top left: Gly290 form H-bond with Ala309; Asn50 form H-bonds with Arg46 (Distance: 2.1Å) and Ala 260 (Distance: 2.8 Å). Top right: The mutant model shows the change of H-bonds, Asn50 form H-bonds with Gly261 (Distance: 2.1 Å) and Ala 260 (Distance: 2.4 Å). Bottom: Wild type and p.Arg46Gln mutation of ACP5. Bottom left: Arg46 form H-bonds with Ser288 (Distance: 1.8 Å and 2.3 Å). Bottom right: The mutant shows the loss of two H-bonds, this might lose the ability to form any polar interaction with other residues. **(F)** Wild type and p.Arg408His mutation of SAMHD1. Left: Arg408 form salt bridge with Asp394. Right: The mutant shows the loss of salt bridge with Asp394, and form H-bond with Ile397 (Distance: 2.3 Å). P, patient; P-M, mother; P-F, father.

The patient was a 2-year-old girl who had recurrent upper respiratory tract infections and oral thrush since birth. She initially presented with cutaneous bleeding spots on both lower extremities, mainly on the calves and dorsum of the feet (Figure 1A). There was no fever, cough, sputum, joint pain, or other signs of discomfort. She was seen at a local pediatric outpatient clinic, which showed thrombocytopenia (4 × 10^9^/L) and decreased hemoglobin (98 g/L). The renal function report showed decreased blood creatinine (Bcr) level (19.3 μmol/L), urine red blood cells (373.4/ul) and urine protein (2+). Serum complement levels were decreased (C3 387 mg/L, C4 44 mg/L). No signs of hematological malignancy were found in bone marrow smear and bone marrow biopsy sections. Systemic lupus erythematosus and idiopathic thrombocytopenic purpura (ITP) were diagnosed based on skin symptoms, thrombocytopenia and decreased serum complement levels. She was then started on methylprednisolone (MP). However, the abnormalities in urine red blood cells and serum complement levels persisted.

Three months later, she presented to our pediatric clinic. Laboratory tests showed elevated white blood cell count (14.63 × 10^9^/L), lymphocyte count (6.45 × 10^9^/L), monocyte count (1.6 × 10^9^/L), calcitonin (0.28 ng/ml), and decreased Bcr (16 μmol/L). Anti-nuclear antibody (ANA, >1/80), anti-ribosomal antibody (ANuA, >1) and anti-dsDNA (31.79 IU/ml) were positive. Serum complement (C3 0.66 g/L, C4 0.06 g/L), immunoglobulin (Ig) A (0.31 g/L) was decreased and IgE (308.47 IU/ml) was increased. The percentage of T lymphocytes (CD3^+^ 35.61%, CD4^+^ 19.78%, CD8^+^ 14.73%) in PBMC was decreased and B lymphocytes were increased (CD19^+^% 57.46%). Mycoplasma pneumoniae IgM antibodies were positive (1:80). Erythrocyte sedimentation rate (ESR), C-reactive protein (CRP) and renal function were normal. Cytomegalovirus (CMV) and Epstein-Barr virus (EBV) antibodies were negative. Rheumatoid factor, IgG, IgM and urine protein were all negative. Antibodies to chlamydial pneumonia, tuberculosis, TORCH, and anti-streptozotocin O were negative. Her abdominal ultrasound scan, skeletal radiography, EEG and brain MRI were normal. Immune markers such as serum immunoglobulin, complement and lymphocyte subpopulation values are listed in Table 1.

**Table 1:** Immune indices of patient.

**Items**	**Patient**	**Reference**
WBC (× 10^9^/L)	14.63↑	3.69–9.16
Hb (g/L)	137	113–151
PLT (× 10^9^/L)	122	101–320
CRP (mg/L)	3.48	0–8
ESR (mm/h)	5	0–20
PCT(ng/mL)	0.280↑	<0.05
Anti-dsDNA(IU/mL)	31.79↑	0–7.0
ANA	1:640 (+)	0
ANuA	4.702 (+)	<1
C3 (g/L)	0.66↓	0.90–1.80
C4 (g/L)	0.06↓	0.10–0.40
IgG (g/L)	11.5	7.00–16.00
IgA (g/L)	0.31↓	0.70–4.00
IgM (g/L)	1.3	0.40–2.30
IgE (IU/mL)	308.47↑	1.31–165
CD3^+^ (%)	35.61↓	61.1–77
CD4^+^ (%)	19.78↓	25.8–41.6
CD8^+^ (%)	14.73↓	18.1–29.6
CD16^+^ CD56^+^ (%)	8.14↓	8.7–38.3
CD19^+^ (%)	57.46↑	4.7–19.3
NK cytotoxic index (%)	8.5↓	15–25
AST(U/L)	42↑	10–40
ALT(U/L)	22	0–75
Urinary protein (mg/24h)	45.1	31–120
EBV-IgG (INDEX)	6.07↑	<1
EBV-IgM (INDEX)	0.08	<1
CMV-IgG (AU/mL)	79 (+)	0–6
CMV-IgM (INDEX)	0.32	0–1

*↑, Increase; ↓, decrease; (+) positive*.

Therefore, she met the diagnostic criteria for SLE and growth retardation (weight: 10.5 kg, −1.20 standard deviation (SD); height: 80 cm, −2.20 SD). She was then started on MP and mycophenolate mofetil (MMF) for 1 year and measures associated with SLE activity remained abnormal (increased dsDNA, positive ANA). At the same time, we found that the patient's IFN-α (31.56 pg/ml), IFN-γ (10.92 pg/ml), TNF-α (8.93 pg/ml), and IL-17A (23.78 pg/ml) increased dramatically during the active phase. Therefore, methotrexate (MTX) was given, but she developed leukopenia (2.76 × 10^9^/L). Considering the inhibitory effect of MTX on bone marrow hematopoiesis, MTX was discontinued at that time and the regimen of MP, HCQ and MMF was changed. However, the patient's indicators such as dsDNA, serum complement and Ig remained unstable.

Given the early onset, multi-organ involvement and the refractory nature of alternating episodes and remissions, we should further explore the etiology to improve the clinical diagnosis and treatment of this patient.

### The Germline Rare Mutations of ACP5 and SAMHD1 Identified in This Patient

Next-generation sequencing brings great convenience for accurate diagnosis. High-throughput sequencing can detect genetic alterations in an entire genome, an exome, or a group of genes. Patients with early-onset lupus often carry a high frequency of pathogenic variants, highlighting the importance of genetic testing for pSLE (21, 22). We then applied WES and identified three new mutations in this patient, all inherited from her father and mother. This patient carried a bilateral missense mutation in the ACP5 gene (NM_001111034.2) at 2 different loci (c.1152G>T [p. Gly290Val, G290V]; c.420G>A [p. Arg46Gln, R46Q]) and a unilateral missense mutation in the SAMDH1 gene (NM_15474.3) (c.1423G>A [p.ARG408HIS, R408H]). The combined annotation-dependent depletion (CADD) scores representing the deleteriousness of these three mutations were 21 (ACP5, c.1152G>T), 22 (ACP5, c.420G>A) and 6.649 (SAMDH1, c.1423G>A) (Figures 1B–D). The sites of these mutations have not been reported previously. Protein structure predictions showed that the G290V mutation resulted in weaker hydrogen bond (H bond) strength, the R46Q mutation in ACP5 resulted in the loss of two H bonds (Figure 1E), and the R408H mutation in SAMHD1 resulted in the loss of the salt bridge and the formation of an H bond (Figure 1F). We know that protein structure is closely related to function, and changes in protein structure caused by mutations may lead to disruption of biological function. Therefore, in combination with the high CADD score and altered protein structure, mutations in ACP5 may have greater pathogenicity.

**Figure 2:**
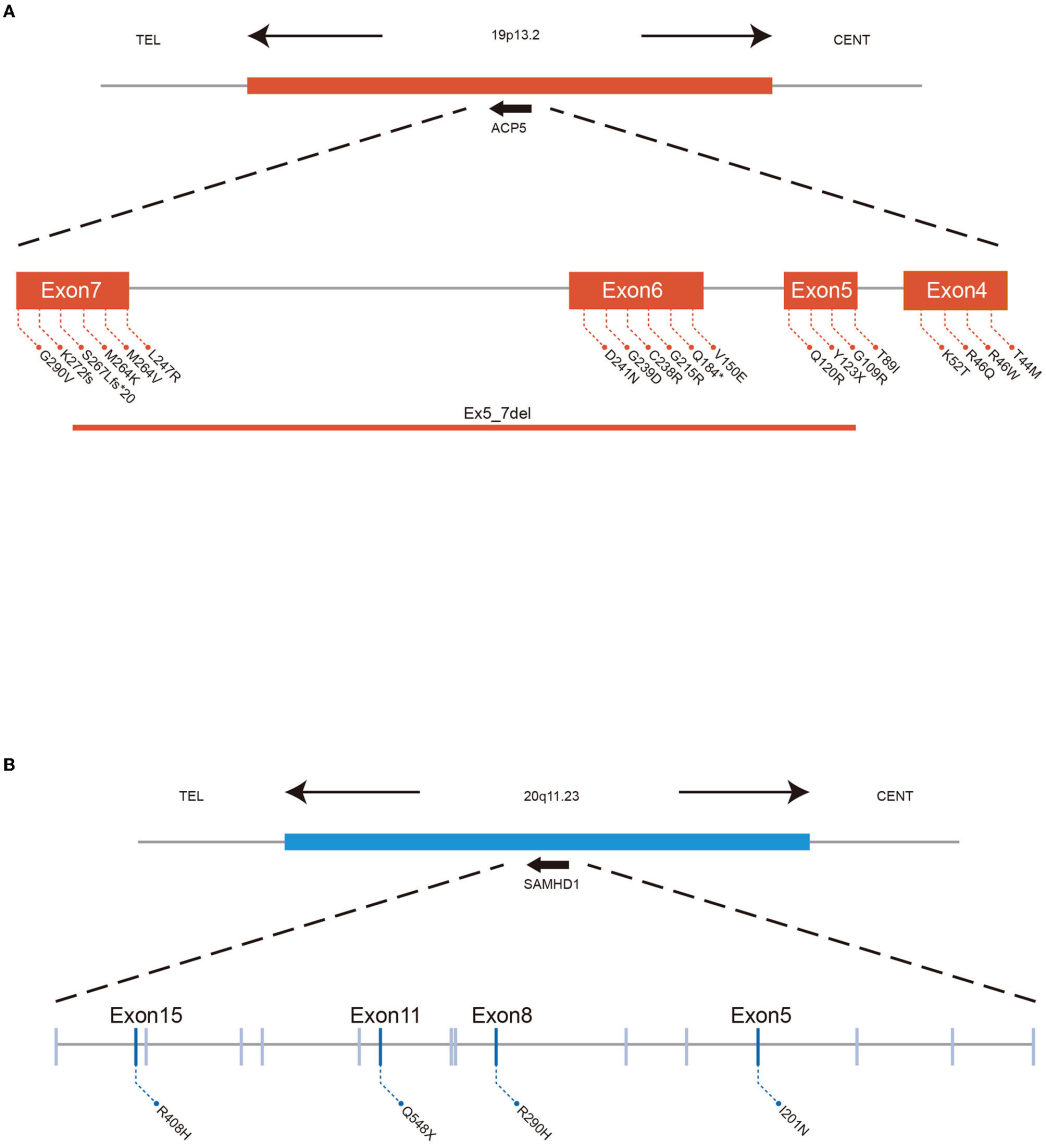
ACP5 mutations cause SPENCD and SAMHD1 mutations cause AGS both diseases clinically overlap with SLE, and both are associated with a spontaneous type I IFN response. **(A)** Summary of ACP5 mutation associated with SLE data. **(B)** Summary of SAMHD1 mutation associated with SLE data.

Mutations in ACP5 and SAMHD1 are known to drive the pathogenesis of SPENCD and AGS, both of which are monogenic interferon diseases characterized by increased type I IFN signaling leading to vasculopathy, autoinflammation, and SLE-like disease (8, 23, 24). To date, 30 cases of SPENCD with SLE have been reported in the literature (23, 25–35) (Figure 2A; Table 2), and 9 cases of SLE with SAMHD1 mutations have been reported in the literature (21, 36–41) (Figure 2B; Table 3), including the patients in this article. Patients with SPENCD or AGS with SLE have a homogeneous clinical presentation, including autoantibody positivity, hematologic involvement, and neurologic symptomatic manifestations. Interestingly, this patient did not show clinical manifestations of epiphyseal lesions of SPENCD and neurological dysfunction of AGS in addition to autoimmune disease, implying that complex protein functional alterations due to polymorphisms in the ACP5 and SAMHD1 genes lead to different clinical symptoms. Here, we used high-throughput sequencing to identify three novel mutations in the coding regions of ACP5 and SAMHD1 in this patient that have not been previously reported to be associated with SLE.

**Table 2:** Cases of SPENCD associated with SLE.

**Case**	**Gender**	**Mutation**	**Amino-acid alteration**	**ANA**	**Anti-dsDNA**	**Thrombocytopenia**	**Proteinuria**	**Hematuria**	**Autoimmunehaemolytic anemia**	**LN**	**Renal involvement**	**Raynaud's /vasculitis**	**Hypocomplementemia (C3 and C4)**	**Drug**	**References**
1	Female	1152G>T/420G>A	G290V Het/R46Q Het	Yes (1:640)	Yes	Yes	No	Yes	No	NA	Yes	Yes	Yes	Prednisolone, HCQ and MMF	
2	Female	369C>A/721G>A	Y123X Het/D241N Het	Yes (1:640)	Yes (1:320)	Yes	Yes	No	No	Class IV	Yes	No	Yes		(20)
3	Female	791 T>A	M264K Hom	Yes (1:1280)	No	Yes	No	No	No	NA	No	No	No		(20)
4	Female	643G>A	G215R Hom	Yes (1:1600)	Yes (>100)	Yes	No	No	Yes	Class V	Yes	No	Yes		(20)
5	Male	325G>A/831 _833delCTA	G109R Het/Y278del Het	Yes	No	Yes	Yes	No	Yes	NA	No	Yes	No	Nivaquin and steroids	(22)
6	Male	791T>A	M264K Hom	Yes	No	No	Yes	No	No	Class V	Yes	No	No		(22)
7	NA	325G>A	G109R Hom	No	No	No	No	No	No	NA	No	No	No		(22)
8	NA	325G>A	G109R Hom	Yes	No	No	Yes	No	No	Class IV	Yes	No	No		(22)
9	NA	155A>C	K52T Hom	Yes	No	Yes	No	No	No	NA	No	No	No		(22)
10	Male	11,543,690-11,548,656 del	Ex5_7 del Hom	Yes (1:640)	Yes (>100)	Yes	Yes	No	No	Class III	Yes	Yes	Yes	Corticosteroids, CQ and CYC	(23)
11	Male	NA	NA	Yes (1:640)	Yes (>100)	No	No	Yes	No	Class IV	Yes	No	Yes	Steroid,CYC,MMF and CQ	(24)
12	Female	NA	NA	Yes (1:320)	Yes (>100)	NA	Yes	No	No	Class V	Yes	Yes	Yes	Steroid,CYC,AZA and CQ	(24)
13	Female	NA	NA	Yes (1:320)	Yes (>100)	No	No	No	No	Class IV	Yes	Yes	Yes	Steroid,CYC and CQ	(24)
14	Male	NA	NA	No	No	No	Yes	Yes	Yes	NA	Yes	Yes	No	Steroid and nivaquine	(25)
15	Female	131C >T/816dupC	T44M Het/K272fs Het	Yes (1:1280)	No	No	Yes	Yes	No	NA	No	Yes	Yes	Steroid,MMF and intravenous Ig	(26)
16	Female	NA	NA	Yes	Yes	No	No	Yes	Yes	NA	No	Yes	Yes	AZA and HCQ	(27)
17	Male	155A >C	K52T Hom	Yes	Yes	No	Yes	No	No	Class II	Yes	Yes	Yes	Prednisolone, AZA, HCQ and rituximab	(27)
18	Male	NA	NA	Yes	Yes	No	Yes	Yes	Yes	Class III	Yes	Yes	NA		(28)
19	Male	449T>A/136C>T	V150E Het/R46W Het	Yes	Yes	No	Yes	No	No	Class IV	Yes	No	Yes	Piroxicam, prednisolone, AZA, gabapentin, topiramate, atenolol and enalapril	(29)
20	Female	449T>A/136C>T	V150E Het/R46W Het	Yes	Yes	No	No	No	No	NA	No	No	Yes	Corticosteroids and infliximab	(29)
]21	Female	550C>T/740T>G	Q184* Het/L247R Het	Yes	Yes	No	No	No	No	IgA nephropathy	Yes	No	No	Amlodipine, enalapril and labetalol	(30)
22	Female	369C >A/721G >A	Y123X Het/D241N Het	Yes (1:640)	Yes (1:320)	No	No	No	Yes	NA	Yes	Yes	No	MMFand prednisolone	(31)
23	Male	266C > T	T89I Hom	Yes (1:640)	Yes (>100)	No	No	No	No	NA	Yes	Yes	No	AZA	(31)
24	Female	791 T>A	M264V Hom	Yes (1:1280)	Yes (>100)	Yes	No	No	Yes	NA	No	No	No		(31)
25	Female	643G>A	G215R Hom	Yes (1:1600)	Yes (>100)	Yes	No	No	Yes	NA	Yes	No	No	Prednisolone, HCQ and MMF	(31)
26	Male	155 A>C/790 A>G	K52T Hom/M264V Het	Yes (1:100)	Yes	No	No	No	Yes	NA	Yes	Yes	No	Prednisolone	(31)
27	Male	359 A>G	Q120R Hom	Yes (1:640)	Yes	No	No	No	Yes	NA	Yes	No	No	Prednisolone	(31)
28	Male	325G>A/712 T>C	G109R Het/C238R Het	Yes (1:640)	Yes	Yes	No	No	Yes	NA	Yes	No	No		(31)
29	Female	131C>T/712 T>C	T44M Het/C238R Het	Yes (1:2560)	Yes	Yes	No	No	Yes	NA	No	No	No		(31)
30	Female	798dupC/716G>A	S267Lfs*20 Het/G239D Het	Yes (1:320)	Yes	Yes	No	No	No	NA	No	No	Yes	Prednisolone and MMF	(32)

*All cases had typical radiographic findings of SPENCD with short stature. Cases and 3, 8 and 9, and 13 and 14, were sib couples. NA, Not assessed/reported; ANA, antinuclear antibodies; LN, lupus nephritis; HCQ, Hydroxochloroquine; MMF, Mycophenolate mofetil; CQ, Chloroquine; CYC, Cyclophosphamide; AZA, Azathioprine*.

**Table 3:** Cases of SLE associated with mutation of SAMHD1.

**Case**	**Gender**	**Mutation**	**Amino-acid alteration**	**ANA**	**Anti-dsDNA**	**Thrombocytopenia**	**Proteinuria**	**Hematuria**	**Autoimmunehaemolytc anemia**	**LN**	**Renal involvement**	**Raynaud's /vasculitis**	**Rash**	**Hypocomplementemia (C3 and C4)**	**Drug**	**References**
**1**	Female	1423G>A	R408H Het	Yes (1:640)	Yes	Yes	No	Yes	No	NA	Yes	No	Yes	Yes	Prednisolone,HCQ and MMF	
**2**	Male	NA		Yes (1:132)	Yes (>100)	Yes	No	Yes	No	NA	Yes	Yes	Yes	Yes	NA	(33, 34)
**3**	Male	NA		No	Yes (>100)	Yes	No	No	Yes	No	No	Yes	Yes	Yes	NA	(33, 34)
**4**	Female	NA		Yes (1:2,500)	Yes (1:140)	Yes	No	No	Yes	No	No	Yes	Yes	No	Prednisolone,AZA	(35)
**5**	Female	NA		NA	NA	Yes	NA	NA	No	NA	NA	No	Yes	No	NA	(36)
**6**	Female	NA		No	Yes	Yes	No	No	No	No	No	No	Yes	No	NA	(36)
**7**	Male	1642C>T/869G>A	Q548X Het/R290H Het	Yes (1:160)	Yes (1:40)	Yes	No	No	Yes	No	No	Yes	Yes	No	Topiramate, intrathecal baclofen, naproxen, etanercept, and prednisone.	(37, 38)
**8**	Female	602T>A	I201N Het	No	No	No	No	No	No	NA	No	Yes	Yes	No	Nifedepine, HCQ	(39)
**9**	Male	602T>A	I201N Het	No	No	No	No	No	No	NA	No	Yes	Yes	No	Nifedepine, HCQ	(39)

*Cases 5 and 6, 8 and 9 were sib couples. NA, Not assessed/reported; ANA, antinuclear antibodies; LN, lupus nephritis; HCQ, Hydroxochloroquine; MMF, Mycophenolate mofetil; AZA, Azathioprine*.

### The Potential Regulation Networks of ACP5 and SAMHD1 in SLE

**Figure 3:**
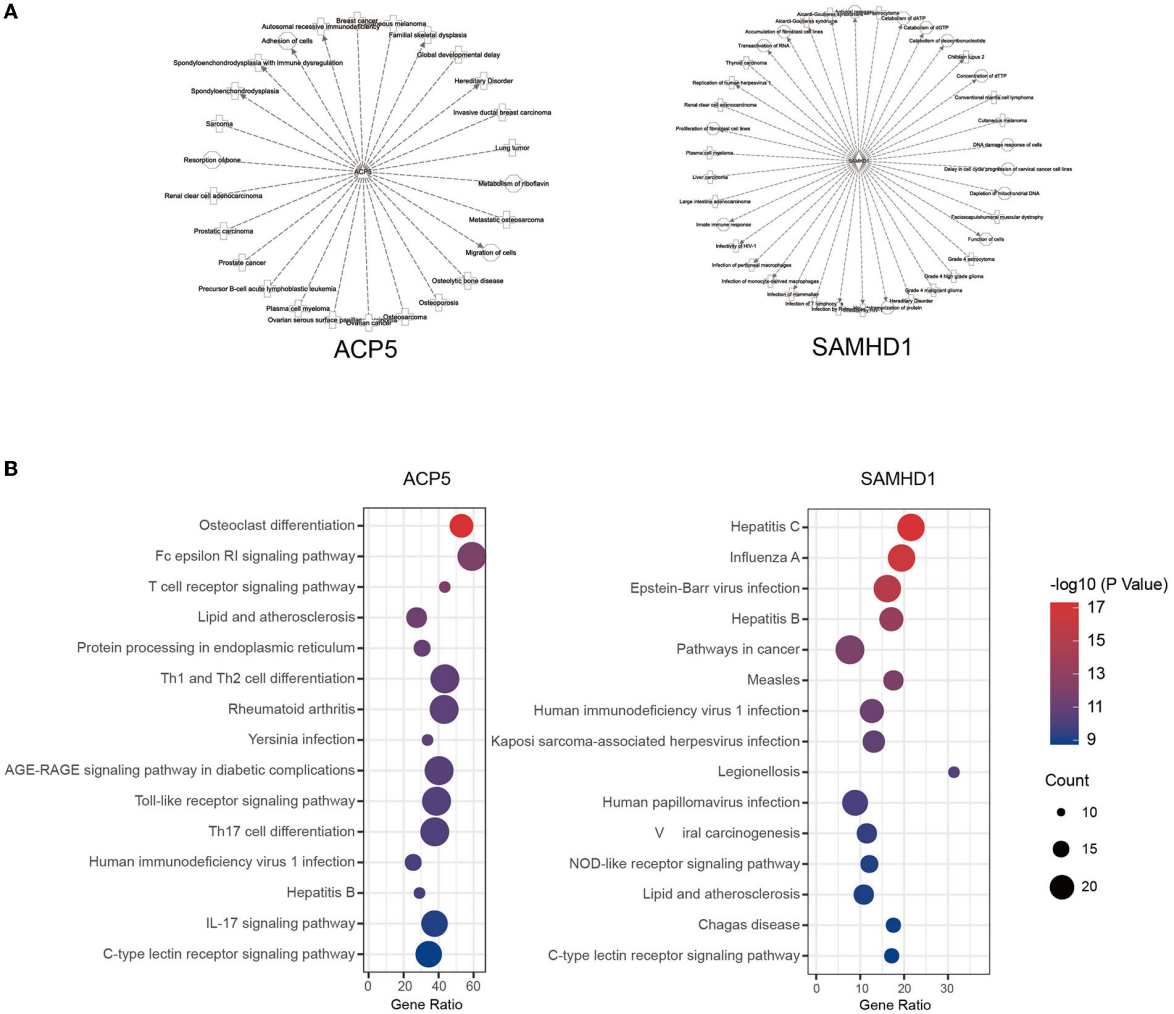
The potential regulation of ACP5 and SAMHD1 in SLE. The phenotype-gene relationship **(A)** and pathway analysis **(B)** were shown to be associated with immunity responses.

To comprehensively summarize these two genes and their potential roles in disease, we used the IPA BioProfiler to explore the phenotype-gene relationships of ACP5 and SAMHD1 (Figure 3A). Indeed, ACP5 is associated with skeletal disorders and other diseases such as neoplastic disorders, including hematological malignancies. The same is true for SAMHD1 with inflammatory, neurological and neoplastic disorders. To further determine the regulatory roles of ACP5 and SAMHD1, we searched the interaction network of these two genes by IPA. We annotated the genome in the network by the online Enrihr tool (Figure 3B). Pathway analysis showed that ACP5 and its interacting genes are associated with osteoclast differentiation, associated with bone disorders in SPENCD. Importantly, many immune pathways were enriched, such as the Toll-like receptor signaling pathway and IL-17 signaling pathway, suggesting a potential regulatory role of ACP5 in SLE, except for type I IFN. For SAMHD1, the enriched pathways mainly were associated with viral infections, implying a dominant effect of genetic alterations in SAMHD1 on infectious diseases. SAMHD1 and its interaction network were associated with the production of type I IFN, as multiple nuclease pathways were significantly associated with it. In conclusion, we found that ACP5 and SAMHD1 are associated with multiple immune pathways that have been shown to be critical in the pathogenesis of SLE, suggesting that genetic perturbations in these two genes may lead to immunological abnormalities in these pathways.

### Interaction of ACP5 and SAMHD1 Is Associated With Interferon Pathway

**Figure 4:**
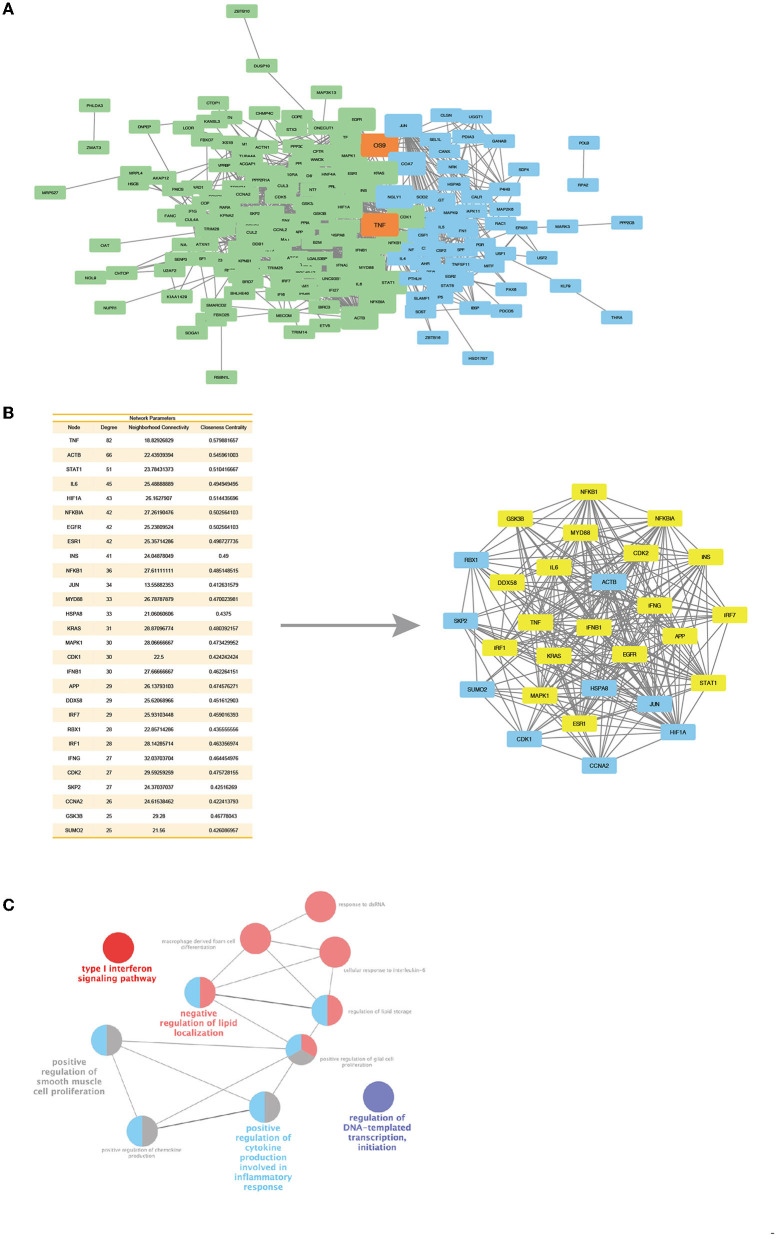
Protein interaction networks of ACP5 and SAMHD1 have connections and a core cluster with significant connections with two networks. **(A)** Merge of ACP5 protein network (green) and SAMHD1 protein network (blue) with overlap nodes in both networks (orange). The size of nodes represents the degree and betweenness of nodes. **(B)** Left: The table outlines the degree, neighborhood connectivity, and closeness centrality values of the core cluster nodes. Right: Networks of core cluster in merge of ACP5 protein network and SAMHD1 protein network. The yellow nodes are the densely connected MCOD clusters, based on their parameters, including connectivity, degree, and centrality. **(C)** ClueGo analysis identifies KEGG pathways and biological processes linked to the essential proteins in the MCOD clusters.

Since ACP5 and SAMHD1 are associated with the type I IFN pathway, which exhibits abnormalities in both SPENCD and AGS. We interrogated the synergistic regulation of these genes by PPI network and pathway analysis and explored whether there is a link between ACP5 and SAMHD1. We constructed two protein interaction networks in Cytoscape to explore the link between ACP5 and SAMHD1 and the potential connection to the etiology of SLE (Figure 4A). The ACP5 protein interaction network included 60 nodes and 277 edges, while the SAMHD1 protein interaction network included 144 nodes and 982 edges. We merged these two networks, with blue representing nodes in the ACP5 protein interaction network and green representing nodes in the SAMHD1 protein interaction network (orange representing nodes in the duplication network). These results demonstrate a significant relationship between these genes (*P*-value for PPI enrichment: <1.0e-16).

Next, we evaluated the central properties of the nodes in the PPI network. Highly significant nodes (score >25) had more interaction partners than non-central nodes, representing proteins with higher relevance in linking regulatory molecules. A unique protein interaction network for ACP5 and SAMHD1 was extracted by overlapping networks, including 28 nodes and 241 edges (Figure 4B). This new network was statistically significant for protein association (PPI-enriched *P*-value: 2.26e-11), showing highly connected edges of both networks, suggesting the possible existence of common pathways involved in the pathogenesis of SLE. To scrutinize the interconnected core clusters of the two protein interaction networks, we identified densely connected MCOD clusters and found an overlap between the ACP5 and SAMHD1 protein networks. The proteins in the core MCOD cluster include IFN-related genes, including MYD88, IRF1, IRF7, and NFKB1. These clusters were also analyzed by KEGG pathway enrichment, including the type I interferon signaling pathway. Collectively, these results further suggest that ACP5 and SAMHD1 can synergistically regulate the type I IFN pathway and that genetic alteration in these two genes accumulate susceptibility to SLE through dysregulation of the IFN pathway (Figure 4C).

## Discussion

**Figure 5:**
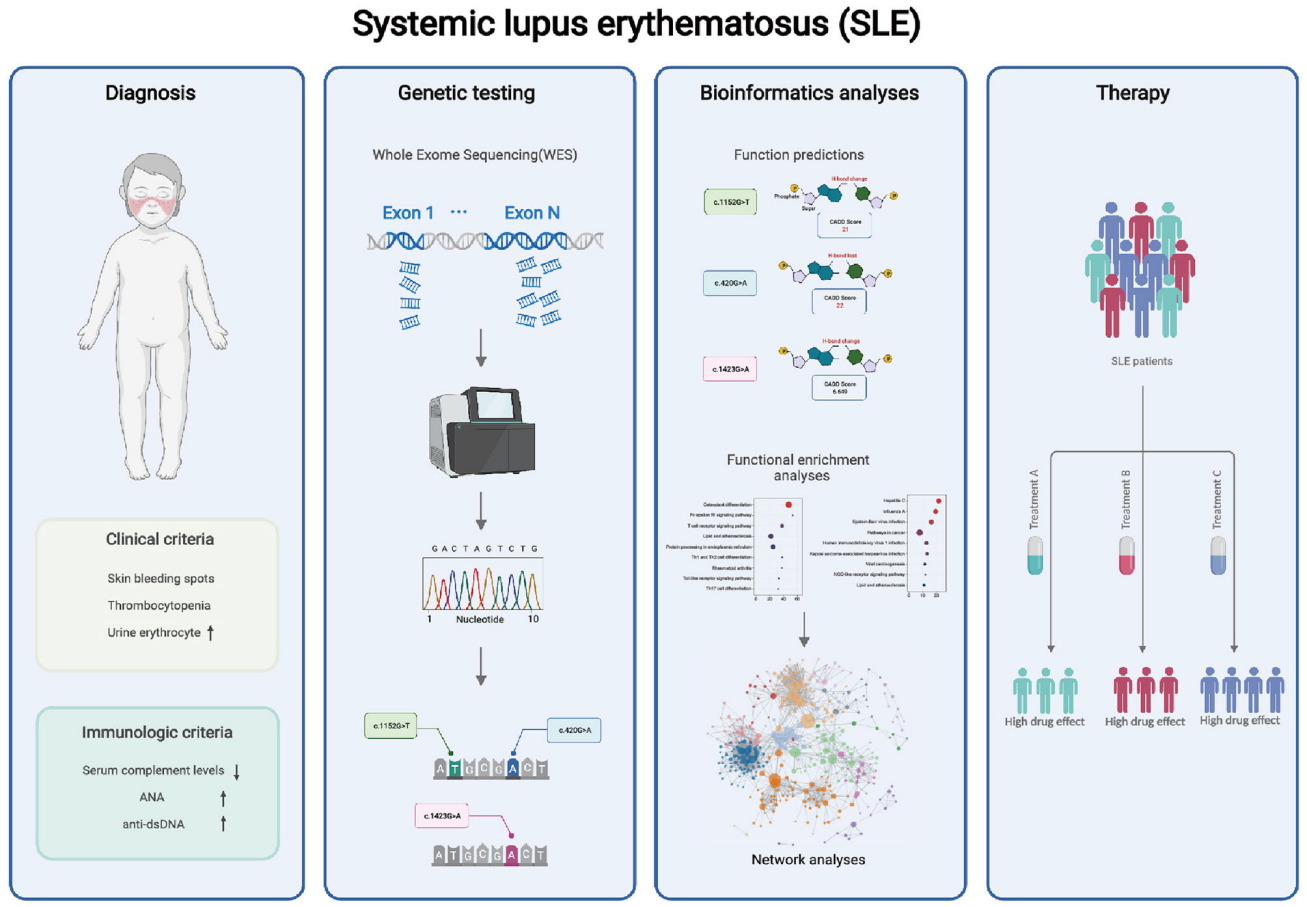
WES may help to analyze the potential pathogenesis of patients. Our findings suggest that the combinatorial complexity of polymorphisms in ACP5 and SAMHD1 coding regions impacts SLE susceptibility, and may contribute to the abnormal immune pathway in SLE. These mutations should be considered for potential precision therapy targeting specific pathways.

In this study, we describe the clinical manifestations of SLE in childhood, with the involvement of organs mainly affecting the skin, kidneys, and hematological system. However, several indicators of SLE remained abnormal after the patient underwent a thorough examination and treatment. We highly suspected that the patient might have some inherited genetic mutations. Then by genome sequencing, we found that the patient had novel missense mutations c.1152G>T and c.420G>A in ACP5 and c.1423G>A in SAMHD1, which were highly predictive of impaired gene function. The further bioinformatic analysis confirmed the regulatory role of these two genes in the type I IFN pathway and revealed that other immune pathways might be affected by mutations in ACP5 and SAMHD1 genes (Figure 5).

Mutations in ACP5 and SAMHD1 genes contribute to the pathogenesis of SPENCD and AGS. Both diseases clinically overlap with SLE and are associated with a spontaneous IFN response (23, 24). However, our patient did not exhibit the typical symptoms of AGS and SPENCD, neither skeletal dysplasia nor neurological dysfunction. Therefore, based on her clinical features, we diagnosed childhood-onset SLE. In addition, we observed a dramatic increase in serum IFN-α during disease flares. The patient presented with refractory SLE, with alternating exacerbations and remissions, and suboptimal treatment outcomes. Identifying genetic defects provides a unique opportunity to apply precise therapies targeting the type I IFN pathway. Unfortunately, our paper has some limitations. We will observe more patients with ACP5 and SAMHD1 mutations by genetic testing and describe the clinical characteristics of these patients.

Through bioinformatic analysis, we found that genetic alterations in ACP5 and SAMHD1 may synergistically enhance the interferon response and participate in the pathogenesis of SLE. However, this patient acquired three germline mutations from her parents. Her mother was normal but carried two mutations in ACP5 and SAMHD1, respectively. Another missense mutation in ACP5 was passed to her by her father. Since these genes are autosomal recessive, the genetic predisposition may come mainly from the double-stranded mutation in ACP5. To clarify whether the pathogenic effect is from a double-copy mutation in ACP5 or a combined defect in ACP5 and SAMHD1 resulting in an enhanced interferon response, we will perform additional functional tests to support our findings directly.

In conclusion, after a comprehensive clinical examination and high-throughput genetic testing, we diagnosed three novel missense mutations in ACP5 and SAMHD1 in a patient with refractory SLE. We analyzed the underlying pathogenesis in this patient, pointing to dysregulated IFN signaling and other indicated immune pathways. These mutations have not been previously reported and should be considered for early and accurate diagnosis of SLE. And the association of dysfunctional APC5 and SAMHD1 with immune abnormalities suggests a potential precise treatment targeting specific pathways.

## Data Availability Statement

The datasets for this article are not publicly available due to concerns regarding participant/patient anonymity. Requests to access the datasets should be directed to the corresponding author.

## Ethics Statement

The studies involving human participants were reviewed and approved by Renji Hospital Biobank is funded by the National Human Genetic Resources Sharing Service Platform (2005DKA21300). Written informed consent to participate in this study was provided by the participants' legal guardian/next of kin. Written informed consent was obtained from the individual(s), and minor(s)' legal guardian/next of kin, for the publication of any potentially identifiable images or data included in this article.

## Author Contributions

S-MH and WC were responsible for article writing, methodology, and genetic interpretation. JF: genetic testing. DD and NS provide writing ideas and guidance. All authors contributed to the article and approved the submitted version.

## Funding

This study was supported by grants from the National Natural Science Foundation of China (31630021, 31930037, 82071843, and 81901637), Shenzhen Science and Technology Project JCYJ20180504170414637 and JCYJ20180302145033769, Shenzhen Futian Public Welfare Scientific Research Project FTWS2021006 and Sanming Project of Medicine in Shenzhen SZSM201602087, Shanghai Municipal Key Medical Center Construction Project (2017ZZ01024-002).

## Conflict of Interest

The authors declare that the research was conducted in the absence of any commercial or financial relationships that could be construed as a potential conflict of interest.

## Publisher's Note

All claims expressed in this article are solely those of the authors and do not necessarily represent those of their affiliated organizations, or those of the publisher, the editors and the reviewers. Any product that may be evaluated in this article, or claim that may be made by its manufacturer, is not guaranteed or endorsed by the publisher.
